# The role of conventional echocardiographic parameters on detecting subclinical anthracycline therapy related cardiac dysfunction—The SATRACD study

**DOI:** 10.3389/fcvm.2022.966230

**Published:** 2022-11-18

**Authors:** Wanzhu Zhang, Feriel Azibani, Elena Libhaber, Joaniter Nankabirwa, Emmy Okello, James Kayima, Isaac Ssinabulya, Karen Sliwa

**Affiliations:** ^1^Department of Medicine and Cardiology, Faculty of Health Science, Cape Heart Institute, University of Cape Town, Cape Town, South Africa; ^2^Department of Adult Cardiology, Uganda Heart Institute, Kampala, Uganda; ^3^Department of Medicine, College of Health Science, Makerere University, Kampala, Uganda; ^4^UMRS 942 Inserm, Paris, France; ^5^Faculty of Health Sciences, School of Clinical Medicine, University of the Witwatersrand, Johannesburg, South Africa; ^6^Infectious Disease Research Collaboration, Kampala, Uganda

**Keywords:** subclinical anthracycline therapy related cardiac dysfunction, mitral annular plane systolic excursion, mitral annular peak systolic tissue Doppler velocity, global longitudinal strain, conventional echocardiography

## Abstract

**Background:**

Subclinical anthracycline therapy related cardiac dysfunction (ATRCD) can be detected with speckle tracking echocardiographic image (STE), which is not widely available in Uganda. We aimed to investigate the role of the two conventional echocardiographic parameters [mitral annular plane systolic excursion (MAPSE) and mitral annular peak systolic tissue Doppler velocity (S’)] on diagnosing subclinical ATRCD.

**Method and results:**

207 cancer patients who underwent anthracycline based chemotherapy were recruited at baseline and followed up until 6 months after ending anthracycline therapy. Comprehensive echocardiographic data were collected at each visit. Global longitudinal strain (GLS) by STE was used as the gold standard diagnostic test to define the case of subclinical ATRCD. Data of the 200 patients who had no evidence of clinical ATRCD were analyzed. One hundred and seventy-two (86.0%) were female, with a median age of 42 years and 47 (23.5%) patients were diagnosed with subclinical ATRCD at the end of anthracycline therapy by GLS criteria. The area under the curve (AUC), cutoff point, sensitivity, specificity, positive predictive value (PPV), and negative predictive value (NPV) of reduction of MAPSE (ΔMAPSE) were 0.6736 (95% CI: 0.5885, 0.7587), ≥ 2 mm, 74.5% (95% CI: 59.7%, 86.1%), 54.9% (95% CI: 46.7%, 63.0%), 33.7% (95% CI: 24.7%, 43.6%), and 87.5% (95% CI: 79.2%, 93.4%). The AUC, cutoff point, sensitivity, specificity, PPV, and NPV of reduction of S’ (ΔS’) were 0.6018 (95% CI: 0.5084, 0.6953), ≥ 0.5 cm/s, 61.7% (95% CI: 46.4%, 75.5%), 52.7% (95% CI: 44.4%, 60.9%), 29.0% (95% CI: 20.4%, 38.9%), and 76.1% (95% CI: 72.4%, 88.6%). When ΔMAPSE and ΔS’ are used as parallel test, the net sensitivity and specificity is 89.4% and 28.8%, respectively, the net PPV and NPV is 27.8% and 90.0%, respectively.

**Conclusion:**

The ΔMAPSE and ΔS’ showed fairly good accuracy, sensitivity and NPV to detect subclinical ATRCD in Ugandan cancer patients. These conventional echocardiographic parameters may serve as screening tools for detecting subclinical ATRCD in resource limited settings.

## Introduction

Anthracyclines is a group highly effective antineoplastic agents used to treat many types of malignancies. Anthracycline therapy related cardiac dysfunction (ATRCD) is the most common and well-recognized chemotherapy induced cardiovascular side effect results from this chemotherapy (1). It is defined as a reduction of left ventricular ejection fraction (LVEF) > 10 percentage point to a value < 50% after exposure to anthracycline (2). The findings of the cotemporary research revealed that anthracycline cardiotoxicity starts with subclinical myocardial cell injury which occurs before the decline of LVEF and symptomatic heart failure. (3, 4). Therefore, detecting ATRCD at subclinical stage will identify the patients at high risk and leads to close monitoring, prompt therapy, and better outcome (3).

Left ventricular Global Longitudinal Strain (GLS) obtained by Speckle Tracking Echocardiography (STE) is currently the most accepted diagnostic tool for subclinical ATRCD (2). It is an accepted and widely used modality for cardio-oncology practice in developed world, but not the case in a low to middle income country. In Uganda, STE is not available in most of the hospital. Moreover, STE requires adequate visualization of the endocardial border and is heavily image quality dependent. This can further limit its application.

When STE technology is not available or not feasible, the assessment of LV longitudinal function by simple conventional ultrasound parameters, namely mitral annular plane systolic excursion (MAPSE) by M-mode echocardiography, or mitral annular peak systolic velocity (S’) by pulsed wave tissue Doppler image (TDI), could be potentially useful tools to provide additional information to LVEF in the evaluation of LV systolic function (5–9). MAPSE and S’ can be easily measured using all the echocardiographic machines and are less dependent on image quality. The current international recommendation is that a progressive decline of MAPSE should raise concern for subclinical LV dysfunction (2). However, no study has defined its cutoff values that allow the prediction of ATRCD. In the present study, we aim to investigate the correlations between GLS and MAPSE/S’, evaluate the roles of MAPSE and S’ on detecting subclinical ATRCD among patients attending Uganda Cancer Institute.

## Materials and methods

**FIGURE 1:**
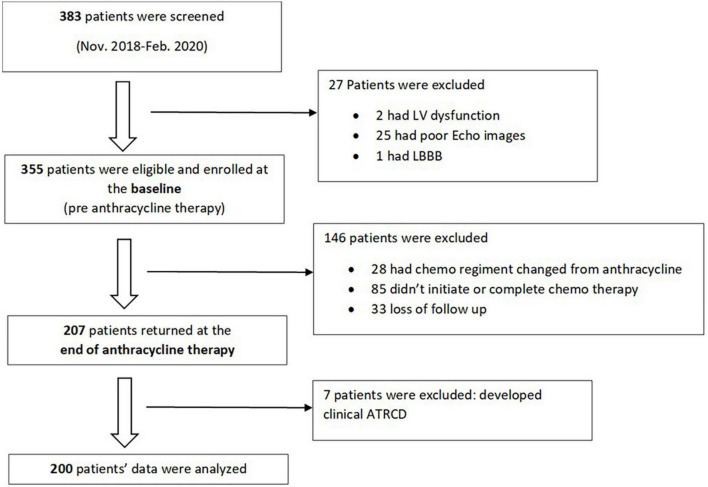
Patients’ flow chart.

We performed a sub analysis of the SATRACD (Detecting subclinical ATRCD in a low-income country) study, which is a cancer cohort who underwent anthracycline based chemotherapy. The SATRACD study design, patient recruitment, and data acquisition have been descripted in details in a published methodology paper (10). In brief, there were 355 patients were recruited at the baseline (pre-chemotherapy) between November 2018 and February 2020. Among them, 207 patients were able to completed anthracycline therapy and being assessed at the end of anthracycline therapy. At the baseline, patient’s demography, cancer profile, and past medical history were recorded. Patients’ symptoms, physical examinations, electrocardiogram (ECG), echocardiographic and laboratory data were collected at baseline and end of anthracycline therapy. Data of the 200 patients who had no evidence of clinical ATRCD at the end of anthracycline therapy were analyzed (Figure 1).

### Definition of subclinical anthracycline therapy related cardiac dysfunction and clinical anthracycline therapy related cardiac dysfunction

American Society of Echocardiography and the European Association of Cardiovascular Imaging criteria (2) were used to define the case of subclinical ATRCD and clinical ATRCD:

Diagnostic criteria for subclinical ATRCD:

• LVEF ≥ 50% and a relative percentage decrease of GLS ≥ 15%, compared with baseline.

Diagnostic criteria for clinical ATRCD:

• Decrease in LVEF of > 10 percentage points, to a value < 50%, compared with baseline.

### Echocardiography protocol and equipment

**FIGURE 2:**
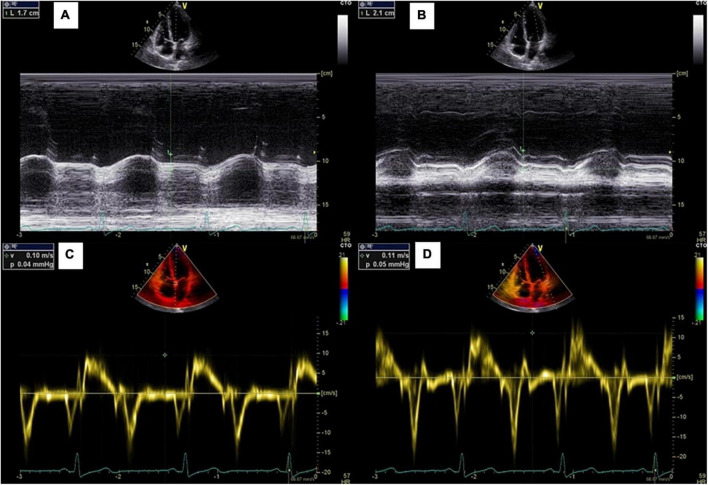
Measurements of mitral annular plane systolic excursion. **(A,B)** Septal and lateral MAPSE measured by M mode echocardiography; **(C,D)** Septal and lateral S’ measured by pulsed-wave tissue Doppler.

Vivid E9^®^ (GE Healthcare) transthoracic echocardiographic machine was used to acquire the cardiac images by two cardiologists with a 1.5–4.6 MHz transducer (M5Sc). All the images were stored and analyzed by a single observer. LVEF was assessed by 2D method (Simpson biplane), from apical 4- and 2-chamber views, calculated by “automatic EF” function. Further manual adjustment was done whenever necessary. MAPSE was measured by M-Mode at the apical 4-chamber view. Septal and lateral MAPSE were obtained and mean value was calculated (Figure 2). S’ was measured by TDI at the apical 4-chamber view. Septal and lateral S’ were obtained and mean value was calculated (Figure 2).

Strain analysis with STE was achieved by applying automatic function imaging (“AFI” function). LV global and regional longitudinal systolic strain were measured through 3 different views (apical 3-, 4-, and 2-chamber views). Manual adjustment was attempted, in the case of unsatisfied endocardial tracking. All the regional longitudinal strains were demonstrated on a Bull’s eye diagram, while GLS was calculated as the mean of the regional longitudinal strains.

We randomly selected 10 patients in order to assess intra and inter-observer reliability. The intraclass correlation coefficients for intra and inter-observer reliability were 0.91 and 0.89, respectively for MAPSE, 0.91 and 0.90, respectively for S’, 0.91 and 0.93, respectively for LV GLS assessments.

### Ethical consideration

The study was approved by School of Medicine Ethics and Research Committee, College of Health Sciences Makerere University (REC REF 2018–081), Uganda National Council of Science and Technology (HS220ES) and Faculty of Health Sciences Human Research Ethics Committee, University of Cape Town (HREC 054/2020sa).

A written copy of the consent was obtained from all participants. Any participants’ question regarding the research was answered prior to signing of the consent form.

### Statistical analysis

We analyzed the data using STATA v14 (Institute Inc., College Station, TX, USA). All continuous variables were expressed as a mean ± standard deviation (*SD*) or medium (interquartile range) and categorical variables as frequencies and percentages. Paired *t*-test and Wilcoxon signed rank test were used to compare dependent continuous variables, where appropriate. Pearson correlation was used to assess the correlation between GLS and MAPSE/S’. Receiver operating characteristic (ROC) analysis was used to assess the accuracy of the two conventional echocardiographic parameters on diagnosing subclinical ATRCD. GLS measured by STE was used as the gold standard diagnostic test to define the case. Empirical cutpoint estimation was done using nearest to (0,1) method. A two-sided *p*-value < 0.05 was considered statistically significant for all analyses.

## Results

In total, 207 patients who were enrolled at the baseline, complete anthracycline therapy and were assessed at the end of the anthracycline therapy. Among them, 7 patients developed clinical ATRCD and were excluded from the data analysis.

The 200 patients who entered data analysis, have a median (IQR) age of 42 (20–69) years, with 172 (86.0%) were female. Patients’ baseline characteristics are showed in Table 1. Breast cancer was the most prevalence cancer (152, 76.0%) in the study population. Other cancer diagnosis includes non-Hodgkin’s lymphoma (15, 7.9%), Hodgkin’s lymphoma (12, 6.4%) and sarcomas (10, 5.3%). Majority of the patients presented with stage 3 (47.6%) and stage 4 (18.7%) diseases. Doxorubicin was the only type of anthracycline received by all the study patients.

**TABLE 1:** Patients baseline characteristics.

Variables	All (*N* = 207)
Age, medium (IQR)	42 (20–69)
Female, *n* (%)	172 (86.0%)
**Physical examination**	
BMI (kg/m2), mean ± *SD*	25.2 ± 4.6
HR, mean ± *SD*	84.8 ± 15.4
SBP (mmHg), mean ± *SD*	130.0 ± 18.0
DBP (mmHg), mean ± *SD*	77.1 ± 11.2
SaO2 (%), mean ± *SD*	97.5 ± 1.9
**Cardiovascular risk factors**	
Hypertension, *n* (%)	46 (23.0%)
DM, *n* (%)	4 (2.2%)
CKD, *n* (%)	2 (1.0%)
HIV positive, *n* (%)	38 (19.0%)
Obesity, *n* (%)	25 (12.5%)
Alcohol use, *n* (%)	34 (17.0%)
Smoking, *n* (%)	1 (0.5%)
**Cancer profile**	
Breast cancer, *n* (%)	152 (76.0%)
Non-Hodgkin’s lymphoma, *n* (%)	15 (7.9%)
Hodgkin’s lymphoma, *n* (%)	12 (6.4%)
Sarcomas, *n* (%)	10 (5.3%)
Stage 1, *n* (%)	27 (14.4%)
Stage 2, *n* (%)	36 (19.3%)
Stage 3, *n* (%)	89 (47.6%)
Stage 4, *n* (%)	35 (18.7%)
Cumulative dose of anthracycline, mg/m^2^ (Mean ± *SD*)	309.8 ± 55.5

BMI, Body mass index; HR, Heart rate; SBP, Systolic blood pressure; DBP, Diastolic blood pressure; SaO^2^, Oxygen saturation.

**FIGURE 3:**
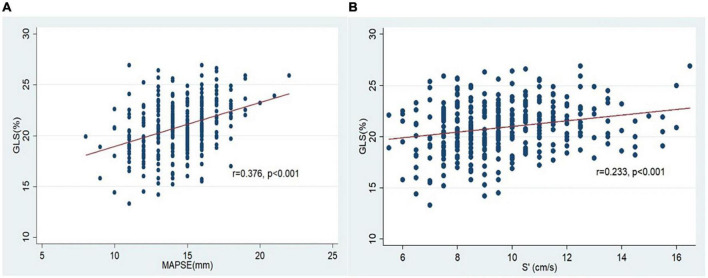
Correlation of GLS and MAPSE/S’ at the baseline. **(A)** Correlation of GLS and MAPSE; **(B)** correlation of GLS and S’.

**FIGURE 4:**
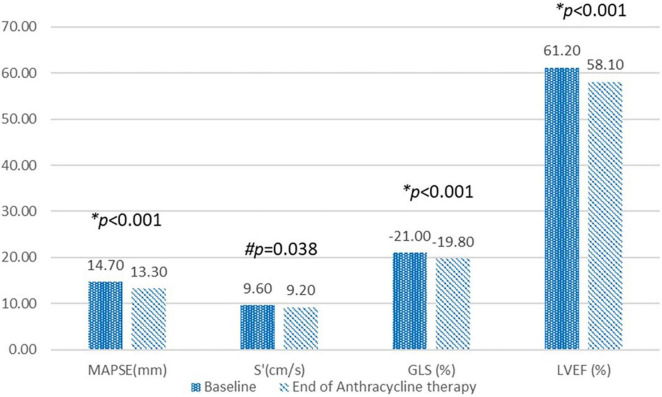
Compare echocardiographic variables at the baseline and at the end of anthracycline therapy. MAPSE, Mitral annular plane systolic excursion; S’, Mitral annular peak systolic tissue Doppler velocity; GLS, Global longitudinal strain; LVEF, left ventricular ejection fraction. *p-value calculated using paired *t*-test. ^#^*p-*value calculated using Wilcoxon signed rank test.

The two conventional echocardiographic parameters- MAPSE and S’, both showed significant positive correlations with the absolute GLS at the baseline (*r* = 0.376, *p* < 0.001 and *r* = 0.233, *p* < 0.001, respectively) (Figure 3). This correlation persisted at the end of the anthracycline therapy (*r* = 0.529, *p* < 0.001 for MAPSE and absolute GLS; *r* = 0.353, *p* < 0.001 for S’ and absolute GLS). At the end of the anthracycline therapy, LVEF, GLS, MAPSE and S’ showed significant reduction compared to baseline measurements (Figure 4). There were 47 (23.5%) patients who were diagnosed with subclinical ATRCD at the end of anthracycline therapy by GLS criteria.

**FIGURE 5:**
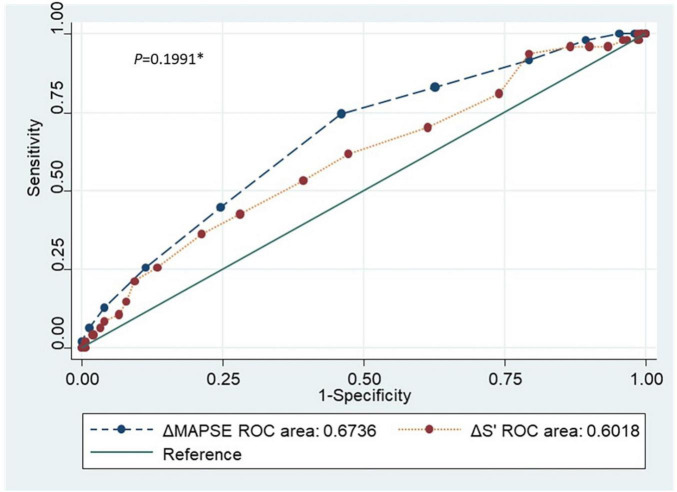
Receiver operating characteristic curve comparing the two conventional echocardiographic parameters (ΔMAPSE and ΔS’). ROC, Receiver operating characteristic; ΔMAPSE, Reduction of mitral annular plane systolic excursion; ΔS’, Reduction of mitral annular peak systolic tissue Doppler velocity. *p-value comparing the accuracy of the 2 conventional echocardiographic parameters.

Figure 5 and Table 2 showed receiver operating characteristic analysis results of the two conventional echocardiographic parameters: Reduction of MAPSE (ΔMAPSE) and reduction of S’(ΔS’). The area under the curve (AUC), cutoff point, sensitivity, specificity, positive predictive value (PPV) and negative predictive value (NPV) of ΔMAPSE were 0.6736 (95% CI: 0.5885, 0.7587), ≥ 2 mm, 74.5% (95% CI: 59.7%, 86.1%), 54.9% (95% CI: 46.7%, 63.0%), 33.7% (95% CI: 24.7%, 43.6%), and 87.5% (95% CI: 79.2%, 93.4%). The AUC, cutoff point, sensitivity, specificity, PPV and NPV of ΔS’ were 0.6018 (95% CI: 0.5084, 0.6953), ≥ 0.5 cm/s, 61.7% (95% CI: 46.4%,75.5%), 52.7% (95% CI: 44.4%, 60.9%), 29.0% (95% CI: 20.4%, 38.9%), and 76.1% (95% CI: 72.4%, 88.6%). When ΔMAPSE and ΔS’ are used as parallel test, meaning both tests are performed in each patient and subclinical ATRCD is considered when any of the test is positive, the net sensitivity and specificity is 89.4% and 28.8%, respectively, the net PPV and NPV is 27.8% and 90.0%, respectively.

**TABLE 2:** ROC analysis for conventional echocardiographic parameters.

Conventional echocardiographic parameters	Cutoff point*	Sensitivity (95% CI)	Specificity (95% CI)	AUC (95%CI)	PPV (95%CI)	NPV (95%CI)
ΔMAPSE (mm)	≥2	74.5% (59.7%, 86.1%)	54.9% (46.7%, 63.0%)	0.6736 (0.5885, 0.7587)	33.7% (24.7%, 43.6%)	87.5% (79.2%, 93.4%)
ΔS’ (cm/s)	≥0.5	61.7% (46.4%, 75.5%)	52.7% (44.4%, 60.9%)	0.6018 (0.5084, 0.6953)	29.0% (20.4%, 38.9%)	76.1% (72.3%, 88.6%)

ΔMAPSE, Reduction of mitral annular plane systolic excursion; ΔS’, Reduction of mitral annular tissue Doppler peak systolic velocity.

*Empirical cutoff point estimated using nearest to (0,1) method.

## Discussion

In the present study we assessed the role of conventional echocardiographic parameters (MAPSE and S’) on detecting subclinical ATRCD. Our results showed fairly good accuracy of both tests.

Subclinical ATRCD has been defined as a relatively reduction of GLS ≥ 15% compare to baseline (2). GLS measured by STI have been proved to be more sensitive than LVEF, as it decreases preceding the significant reduction of LVEF in patients treated with anthracycline therapy (11, 12). GLS represents the LV longitudinal systolic function that is the most important component of LV global systolic function. Other components of LV systolic function include radial contraction and circumferential twisting (13).

Left ventricular longitudinal function can be reduced despite of a normal LVEF, and this has been demonstrated using several echocardiographic parameters including GLS by STE (14–17), MAPSE by M-mode (18, 19) and S’ by TDI (20–23). MAPSE, representing the longitudinal motion of the mitral annular plane, is the distance of displacement of mitral annular between the end diastole and peak systole. S’ obtained by TDI is another parameter that represents the longitudinal motion of the mitral annular plane. Deferent from MAPSE, S’ is the peak systolic velocity of the mitral annulus.

As a novel echocardiographic modality, GLS has several advantages over MAPSE and S’ such as its relative independence from translation, tethering and the angle of incidence (24). However, an accurate GLS measurement depend on first, having non-foreshortened LV imaging in all three apical LV views, second, a good image quality with adequate endocardial tracking in the most of the 6 segments in each of the apical views (24). In fact, in large studies in subjects with normal LVEF, an accurate GLS measurement has not been possible in a significant proportion of the subjects (16, 17, 25). In our study group, GLS measurement was not feasible in 25/383 (6.5%) patients at the initial screening stage. In contrast, MAPSE and S’ could be measured in all subjects in the present study. This consistent with the reported high feasibility of MAPSE and S’ measurements in population studies (26–29). On the other hand, the limited availability of STE has been the biggest hinder of its usefulness in Uganda. The present study compared the two more feasible echocardiographic parameters (MAPSE/S’) with GLS to assess their role on detecting subclinical ATRCD.

We first assessed the correlation between GLS and MAPSE/S’. The presence of positive correlations of GLS with MAPSE and S’ in our study are also reported in the previous studies (16, 17, 19, 24, 30). The observation of a closer relationship between GLS and MAPSE (*r* = 0.376) compared to that between GLS and S’(*r* = 0.233) was also observed by Peverill and Cheng (24). This may be explained by MAPSE being a distance parameter, can be a better surrogate of GLS—a parameter of relative change of myocardial length, than S’ which is a velocity parameter.

To define the ability of MAPSE/S’ to detect subclinical ATRCD, ROC analysis was done to compare these conventional echocardiographic parameters with GLS. Considering MAPSE, S’, and GLS have similar biological variability (declined with age) (27, 28) and ΔGLS is used in the guideline (2), we studied ΔMAPSE/ΔS’ as the variables of the test to be investigated. Our results showed fairly good accuracy on both ΔMAPSE and ΔS’.

Several studies demonstrated the usefulness of S’ in detecting subclinical LV systolic dysfunction in deferent clinical settings. Study published by Vinereanu et al. in 2003 and Raafat et al. in 2018 suggested measurement of S’ may be a more sensitive marker of subclinical changes in LV performance in diabetes than assessment of global function by LVEF (23, 31). However, both studies didn’t compare S’ with GLS. In the setting of cancer population, Fallah-Rad et al. enrolled 42 breast cancer patients who underwent anthracycline plus trastuzumab therapy. They found a significantly reduced S’ (cutoff of 0.6 cm/s) detected as early as 3 months after chemotherapy seems to predict a decline in LVEF after 6 months with high sensitivity (93%) and specificity (99%) (32). This result is very close to our study finding regarding the S’ cutoff of 0.5 cm/s. The different study methods could have contributed to the different sensitivity and specificity of S’, since we used GLS as the reference test for assessment but not the case in Nazanin Fallah study, which compared S’ with LVEF. Zhang et al. investigated 82 patients with diffuse large B-cell lymphoma treated with anthracycline based chemotherapy and reported S’ < 13.65 cm/s (sensitivity, 66.7%; specificity, 71%; AUC = 0.682) after 2–4 chemotherapy cycles from the baseline values can reliably predict cardiotoxicity (33). Although the accuracy of S’ in this study is similar with our study, however, other than our study that ΔS’ was tested, they tested the value of S’. There is also report of greater sensitivity of S’ compared with LVEF for detection of early anthracycline toxicity by Florescu group (34), but no cutoff value of S’ was defined in this study.

Previous studies have also demonstrated the role of MAPSE in various cardiovascular diseases. Reduced MAPSE can be used as a sensitive early marker of LV systolic dysfunction in hypertension (35), diabetes (36), coronary artery disease (37), and aortic stenosis (38–40). MAPSE has been described as a useful and easily acquired measurement, especially on exercise, for the diagnosis of heart failure patients with preserved EF (19). Present study assessed the role of MAPSE in detecting subclinical ATRCD, an area has received little attention in previous studies.

We found ΔMAPSE ≥ 2 mm was able to detect subclinical ATRCD with AUC of 0.6736, sensitivity of 74.47% and specificity of 54.9%.

It is to be noted our results showed both ΔMAPSE ≥ 2 mm and ΔS’ ≥ 0.5 cm/s had a higher NPV (87.5 and 76.1%) than PPV (33.7 and 29.0%), which underlining these parameters can serve as a screening test for subclinical ATRCD when GLS by STE is not available or feasible. The sensitivity and NPV are further improved when the two test are used as simultaneous (parallel) test.

### Limitations

Our study is so far the largest anthracycline therapy cohort in the country to investigate the accuracy of conventional echocardiographic parameters to detect subclinical ATRCD. Nevertheless, it needed to note a major limitation of MAPSE/S’ is they are not able to show the LV regional longitudinal function, while GLS by STE can easily demonstrate this information by the bull’s eye diagram (41, 42). Besides, some degree of potential bias could be caused by the fact that the echocardiographic observers were not blinded to the patients’ status.

## Conclusion

MAPSE and S’ have a significant correlation with absolute GLS. The ΔMAPSE and ΔS’ showed fairly good accuracy, sensitivity and NPV to detect subclinical ATRCD in Ugandan cancer patients. These simple conventional echocardiographic parameters may serve as screening tools for detecting subclinical ATRCD in resource limited settings where GLS by STI is not available or not feasible. Both test maybe measured simultaneously whenever possible in order to improve the sensitivity and NPV.

## Data availability statement

The raw data supporting the conclusions of this article will be made available by the authors, without undue reservation.

## Ethics statement

The studies involving human participants were reviewed and approved by the School of Medicine Ethics and Research Committee, College of Health Sciences Makerere University. The patients/participants provided their written informed consent to participate in this study.

## Author contributions

WZ, KS, FA, EO, and JK contributed to conception and design of the study. WZ and IS organized the database. WZ, EL, and JN performed the statistical analysis. WZ wrote the first draft of the manuscript. All authors contributed to manuscript revision, read, and approved the submitted version.
